# Restoring Quality of Life: A Comprehensive Review of Penile Rehabilitation Techniques Following Prostate Surgery

**DOI:** 10.7759/cureus.38186

**Published:** 2023-04-27

**Authors:** Prathvi S Thakur, Pankaj Gharde, Roshan Prasad, Mayur B Wanjari, Ranjana Sharma

**Affiliations:** 1 Department of Surgery, Jawaharlal Nehru Medical College, Datta Meghe Institute of Higher Education and Research, Wardha, IND; 2 Department of General Surgery, Jawaharlal Nehru Medical College, Datta Meghe Institute of Higher Education and Research, Wardha, IND; 3 Department of Medicine and Surgery, Jawaharlal Nehru Medical College, Datta Meghe Institute of Higher Education and Research, Wardha, IND; 4 Department of Research and Development, Jawaharlal Nehru Medical College, Datta Meghe Institute of Higher Education and Research, Wardha, IND; 5 Department of Medical Surgical Nursing, Smt. Radhikabai Meghe Memorial College of Nursing, Datta Meghe Institute of Higher Education and Research, Wardha, IND

**Keywords:** radical prostatectomy, quality of life, erectile dysfunction, prostate surgery, penile rehabilitation

## Abstract

Prostate cancer is the most common type of cancer in men, and its treatment options include surgery, radiation therapy, and chemotherapy. Prostate surgery can often result in erectile dysfunction (ED), significantly impacting patients' quality of life. Penile rehabilitation techniques have been developed to restore erectile function following prostate surgery. This review discusses the different penile rehabilitation techniques available, their effectiveness, and the factors affecting their success. This paper also addresses the importance of addressing the psychological aspects of ED in these patients and the need for personalized and tailored rehabilitation plans. By providing a comprehensive understanding of penile rehabilitation techniques, this paper can assist clinicians in restoring the quality of life of patients who have undergone prostate surgery.

## Introduction and background

Prostate cancer is the most diagnosed cancer in men worldwide, with over 1.4 million new cases reported annually [1]. Radical prostatectomy (RP) is a widely used treatment for prostate cancer, with over 300,000 procedures performed annually [2]. While RP is an effective treatment for prostate cancer, it can have a significant impact on a man's sexual function, including erectile dysfunction (ED). Up to 80% of men experience ED following RP, with around 50% of men reporting severe or complete ED [3,4].

Penile rehabilitation is an essential aspect of postoperative care for men who have undergone RP. Penile rehabilitation involves the use of various techniques to improve erectile function following surgery. The primary goals of penile rehabilitation are to enhance the quality of erections, restore sexual function, and improve the patient's quality of life [5]. The importance of penile rehabilitation following RP is well-established, with numerous studies showing that early intervention can lead to improved outcomes [6,7].

The objective of this paper is to provide a comprehensive overview of the current state of knowledge regarding penile rehabilitation techniques following prostate surgery. This review will focus on the most used penile rehabilitation techniques, including oral medications, penile injections, and vacuum devices. We will examine the timing of penile rehabilitation interventions and the outcomes measured, including erectile function, quality of life, and patient satisfaction. This review aims to provide an updated and comprehensive overview of the current evidence on penile rehabilitation techniques following RP.

## Review

Methodology

**Figure 1:**
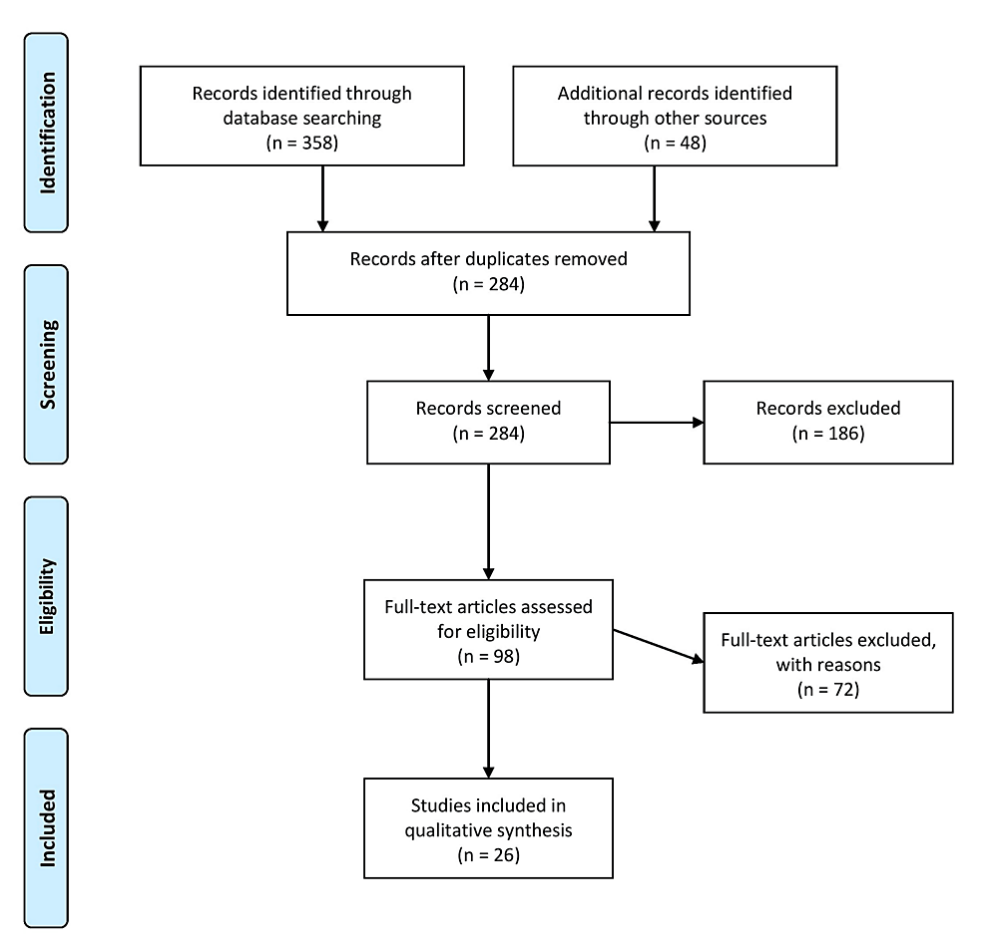
The selection process of articles used in this study. Adopted from the Preferred Reporting Items for Systematic Reviews and Meta-Analyses (PRISMA).

The search was conducted using electronic databases, including PubMed, MEDLINE, and Cochrane Library, using medical subject headings (MeSH) with keywords, "radical prostatectomy", "quality of life", "erectile dysfunction", "prostate surgery", and "penile rehabilitation." Inclusion criteria included studies written in English, peer-reviewed papers published between 2000 and 2022, and in vivo human studies reporting on penile rehabilitation techniques following prostate surgery. Exclusion criteria were animal studies, case reports, and studies reporting on nonsurgical interventions for ED. Figure 1 describes the selection process of articles used in our study.

Description of prostate surgery and its impact on erectile function

Prostate cancer is a prevalent malignancy, and radical prostatectomy is one of the most common surgical interventions used to treat it. While this procedure has shown efficacy in eradicating the cancerous tissue, it can potentially engender deleterious consequences on sexual function, chiefly erectile function. The prostate gland is proximal to nerves and blood vessels that innervate and perfuse the penis, respectively, and its excision or damage during surgery can culminate in ED [8]. It is imperative to note that the impact of prostate surgery on erectile function is multifactorial, contingent on various parameters, such as the patient's age, comorbidities, and the scope of the surgical intervention. Notwithstanding these confounding factors, empirical data suggests that up to 80% of men experience ED following prostatectomy [9].

Overview of the existing literature on penile rehabilitation techniques following prostate surgery

Prostate surgery is known to have a negative impact on erectile function, leading to significant interest in developing penile rehabilitation techniques to aid men in recovering their erectile function postsurgery. Penile rehabilitation encompasses a variety of therapies, including oral medications, penile injections, vacuum devices, and other interventions aimed at increasing blood flow and stimulating nerve growth in the penis. Innumerable studies have investigated the efficacy of such techniques following prostate surgery [10,11].

The success of penile rehabilitation is influenced by various factors, including the timing of the intervention, the type of rehabilitation technique utilized, and the degree of ED experienced by the patient. Some studies have indicated that early penile rehabilitation, conducted within a few weeks of surgery, proves to be more effective than delayed rehabilitation [12,13].

Penile rehabilitation techniques

Oral Medications

Phosphodiesterase type 5 inhibitors (PDE5i), namely, sildenafil, tadalafil, and vardenafil, are orally administered medications employed for penile rehabilitation following prostate surgery. These compounds impede the degradation of cyclic guanosine monophosphate (cGMP), a crucial mediator that facilitates the relaxation of the smooth muscles and escalation of blood flow to the penile tissue. The aforementioned mechanisms of action of PDE5i render them a potentially effective treatment for the enhancement of erectile function and preservation of penile health after prostate surgery. Nonetheless, it is worth noting that the efficacy of PDE5i is not universal, and some individuals may suffer from side effects such as headaches, flushing, or gastrointestinal disturbances. Furthermore, using PDE5i concurrently with certain medications, such as nitrates, is contraindicated, as this could result in an alarming reduction in blood pressure [10].

Penile Injections

Penile injections refer to administering medications such as alprostadil or papaverine via direct injection into the penis, which aims to induce enhanced blood flow and promote erectile function. This technique is often resorted to when conventional oral medications fail to yield the desired results or are not tolerated by the patient. While penile injections have demonstrated efficacy in improving erectile function, they can also potentially result in adverse effects such as pain, bruising, or scarring at the injection site. Hence, patients must receive adequate training on the appropriate injection techniques and adhere to strict guidelines to minimize the risk of infection or other complications [14].

Vacuum Devices

Vacuum devices, specifically penile pumps, establish a negative pressure environment around the penis, which causes blood to flow into the erectile tissue. Afterward, the device is removed, and a constrictive band is positioned at the penis base to uphold the erection. These vacuum devices can be implemented as a monotherapy or combined with other treatments. While vacuum devices are generally perceived as safe and efficacious, they may be accompanied by adverse effects such as bruising or numbness. Additionally, it is crucial to handle the constriction band with caution to prevent tissue injury or other associated complications [15].

Other Penile Rehabilitation Techniques

Additional penile rehabilitation techniques that have been the subject of research include low-intensity shockwave therapy, penile vibratory stimulation, and penile traction therapy. Low-intensity shockwave therapy entails utilizing low-energy shockwaves to stimulate blood flow and encourage tissue repair in the penis. Penile vibratory stimulation involves a device that vibrates the penis to boost blood flow and promote nerve regeneration. Penile traction therapy requires a device that gently applies traction to the penis to enhance tissue growth and increase penile length [16,17].

Although these techniques have displayed promise in initial studies, further investigation is necessary to establish their efficacy and optimal employment in penile rehabilitation after prostate surgery. Table 1 summarizes the various techniques, their mechanism of action, and their adverse effects.

**Table 1:** Summary of the various penile rehabilitation techniques available, their mechanisms of action, and their adverse effects. The author has recreated from [10,14,15]. PDE5i, phosphodiesterase type 5 inhibitors; cGMP, cyclic guanosine monophosphate; GI, gastrointestinal

Technique	Mechanism of Action	Adverse effects
Oral medication	These mainly have PDE5i, which works by preventing the degradation of cGMP, whose higher levels increase the blood flow to the penile tissue and also relax the erectile tissue.	Side effects, such as headache, flushing, and GI disturbances, vary from person to person. Also, it cannot be used with nitrates, as it risks the patient for hypotension.
Penile injections	The injections contain medicines that increase the blood flow to the penile tissue, thus upholding the erection.	Patients need to get trained for self-administration of the injections, or else they can suffer pain, bruising, or scarring at the injection site.
Vacuum devices	Creates a negative pressure around the penis, due to which blood flow to the erectile tissue increases, followed by positioning of a constrictive band at the base of the penis to maintain the erection.	The pumps can cause bruising or numbness if the pressure gets too low. Also, the constriction band can injure the tissue if it gets too tight, leading to other complications.

Outcomes of penile rehabilitation

Erectile Function

The primary outcome measure in most studies on penile rehabilitation following prostate surgery is erectile function. Several studies have reported significant improvements in erectile function with penile rehabilitation techniques, including PDE5i, vacuum erection devices, and penile injections. One randomized controlled trial found that patients who received PDE5i therapy beginning four weeks after surgery had significantly higher rates of erectile function recovery than those who received a placebo [18]. Another study found that vacuum erection devices used three times per week for 20 minutes at a time resulted in significant improvements in erectile function [18-20].

Quality of Life

Prostate surgery is a widely utilized treatment for prostate cancer. However, it can significantly impact patients' quality of life, as it often leads to ED. Penile rehabilitation techniques, such as PDE5i, have emerged as promising therapies to mitigate this complication and enhance patients' overall quality of life following prostate surgery. The benefits of penile rehabilitation extend beyond sexual function. For instance, a study reported that patients who underwent rehabilitation had significantly improved urinary function and overall satisfaction with their sexual and urinary health compared to those who did not [6-9].

Incorporating penile rehabilitation into the postoperative care of patients undergoing prostate surgery could potentially enhance their quality of life and overall recovery. These findings underscore the importance of addressing the sexual and urinary health of patients undergoing prostate surgery and highlight the potential benefits of penile rehabilitation techniques in achieving this goal [21,22].

Patient Satisfaction

Patient satisfaction is a critical outcome measure for evaluating the success of penile rehabilitation following prostate surgery. After prostate surgery, many men may experience ED, which can significantly affect their quality of life. Measuring patient satisfaction following penile rehabilitation involves assessing various aspects of the patient's experience, including their level of satisfaction with the treatment, the degree of improvement in their erectile function, and the impact on their overall sexual function and quality of life [14-16].

Patient satisfaction surveys can gather information about these aspects of the patient experience. These surveys may include questions about the patient's level of satisfaction with the treatment they received, the degree of improvement in their erectile function, and any adverse effects or complications they experienced. By measuring patient satisfaction, healthcare providers can better understand the effectiveness of their penile rehabilitation programs and make necessary adjustments to improve patient outcomes. In addition, patient satisfaction can be a valuable tool for guiding treatment decisions and improving patient education and support [18,21-23].

Clinical implications

Recommendations for Clinical Practices

Based on the existing literature, it is recommended that men undergoing prostate surgery receive penile rehabilitation to help preserve and potentially restore erectile function. PDE5i are one of the most commonly used penile rehabilitation techniques and be effective in improving erectile function following prostate surgery [20,24].

In addition to PDE5i, other penile rehabilitation techniques, such as penile injections, vacuum devices, and penile vibratory stimulation, may also be effective. The exact timing and duration of penile rehabilitation are still being studied, but early intervention (within a few weeks of surgery) proves to be more effective than delayed intervention [11,25]. Healthcare providers must educate patients about the potential impact of prostate surgery on sexual function and the importance of penile rehabilitation. Patients should be given information on the various penile rehabilitation techniques and their potential benefits and risks.

Potential Barriers to Implementation

Despite the potential benefits of penile rehabilitation, several barriers exist to implementation in clinical practice. One barrier is healthcare providers' lack of awareness and education about the importance of penile rehabilitation and the available techniques. This can lead to underutilizing penile rehabilitation and a missed opportunity to improve a patient's quality of life. Another barrier is the cost of penile rehabilitation techniques, particularly for patients without insurance coverage or limited resources. Some patients may be reluctant to use certain techniques due to perceived invasiveness or inconvenience [26].

Future Research Directions

While the existing literature suggests that penile rehabilitation can effectively improve erectile function following prostate surgery, there is still much to learn about the optimal timing, duration, and type of penile rehabilitation. Future research should focus on addressing these questions to improve outcomes for patients. There is also a need for more studies on the long-term effects of penile rehabilitation, including its impact on quality of life and overall sexual function. Additionally, research is needed on the potential benefits of combining different penile rehabilitation techniques for optimal outcomes.

## Conclusions

In conclusion, prostate surgery is a common treatment for prostate cancer but can have a significant impact on erectile function. Penile rehabilitation techniques, such as PDE5i, penile injections, vacuum devices, and penile vibratory stimulation, can be effective in improving erectile function following surgery. Early intervention may be more effective than delayed intervention, and healthcare providers should educate patients about the importance of penile rehabilitation. While there are potential barriers to implementation, such as the lack of awareness and education among healthcare providers and cost considerations, it is important to prioritize penile rehabilitation in clinical practice to improve patients' quality of life. Future research should focus on addressing questions about the optimal timing, duration, and type of penile rehabilitation, as well as the long-term effects of these interventions. Overall, penile rehabilitation is a promising approach to preserving and restoring erectile function following prostate surgery and should be considered an important aspect of patient care.
